# Clinical comparative study of glasses-free 3D and 2D thoracoscopic surgery in minimally invasive esophagectomy

**DOI:** 10.3389/fonc.2022.959484

**Published:** 2022-08-05

**Authors:** Rongqiang Wei, Xinyu Ding, Zihao Chen, Ning Xin, Chengdong Liu, Yunhao Fang, Zhifei Xu, Kenan Huang, Hua Tang

**Affiliations:** ^1^ Department of Minimally Invasive Thoracic Surgery Center, Changzheng Hospital, Naval Medical University, Shanghai, China; ^2^ Department of Thoracic Surgery, The First Affiliated Hospital of Soochow University, Suzhou, China

**Keywords:** glasses-free 3D display system, thoracoscopy, minimally invasive esophagectomy (MIE), esophageal squamous cell carcinoma, thoracic surgery

## Abstract

**Objective:**

To investigate the safety and efficacy of glasses-free three-dimensional (3D) thoracoscopic surgery in minimally invasive esophagectomy (MIE).

**Methods:**

The clinical data of 98 patients, including 81 men and 17 women aged 45–77 years, with esophageal squamous cell carcinoma who underwent minimally invasive thoracoscopic esophagectomy from January 2017 to December 2019 [3 years, with clinical follow-up time: 1 year~4 years (2017.01–2020.12)] were retrospectively analyzed. Patients were divided into two groups according to different surgical methods including a glasses-free 3D thoracoscopic group (G-3D group: 38 patients) and a two-dimesional (2D) thoracoscopic group (2D group: 60 patients). The clinical outcome of the two groups were compared.

**Results:**

The operation time of the thoracoscopic part in the G-3D group was significantly shorter than that in the 2D group (P<0.05). The total number of lymph node dissection in the G-3D group was more than that in the 2D group (P<0.05). The thoracic indwelling time, postoperative hospital stay, severe pulmonary infection, arrhythmia, anastomotic leakage, chylothorax, and recurrent laryngeal nerve injury were not significantly different between the two groups (P>0.05). There was also no significant difference between the two groups on the progression-free survival (P>0.05).

**Conclusion:**

Glasses-free 3D thoracoscopic surgery for esophageal cancer is a safe and effective surgical procedure. Compared with 2D thoracoscopic MIE, glasses-free 3D thoracoscopic MIE for esophageal cancer has higher safety, more lymph node dissection, and higher operation efficiency through the optimized surgical operations. We believe that glasses-free 3D thoracoscopy for MIE is worthy of clinical promotion.

## Introduction

Esophageal cancer is a common malignant tumor of the digestive tract. When diagnosed, most of the patients are in the advanced stage and lose the chance of surgery, whereas for some early-stage patients, surgery is the preferred treatment procedure (1, 2). With the development of minimally invasive technology, minimally invasive surgery for esophageal cancer becomes more mature and has gradually been recognized (3–6). From the earliest thoracoscopy combined with laparotomy or laparoscope combined with thoracotomy, the surgery procedure for esophageal cancer radical resection is gradually transited to thoracoscopy combined with laparoscopy. However, the minimally invasive techniques or equipment, such as two-dimensional (2D) thoracoscopy or three-dimensional (3D) thoracoscopy, still have some problems at present. Compared with 2D thoracoscopy, 3D thoracoscopy can improve the comfort of surgeons’ intraoperative performing by being provided with the 3D structure of the target tissues and organs, which can effectively avoid accidental injury and bleeding during the operation (7–9). However, because of the long operation time, special 3D imaging technology, and the darker visual effect of surgical field during performing 3D thoracoscopic esophagectomy, some surgeons wearing 3D glasses are prone to dizziness, asthenopia, visual ghosting, or other discomfortable symptoms (10, 11). In 2015, the world’s first glasses-free 3D thoracoscopic surgery was completed in Guangzhou, China (12). Glasses-free 3D technology may solve the problems to a certain extent; our study performed domestic glasses-free 3D thoracoscopic and 2D thoracoscopic surgery for minimally invasive esophagectomy (MIE) in our hospital from January 2017 to December 2019. The objective was to evaluate the advantages and disadvantages of domestic glasses-free 3D thoracoscopy in esophageal cancer surgery and provide guidance for clinical practice.

## Materials and methods

### Patients

**Figure 1:**
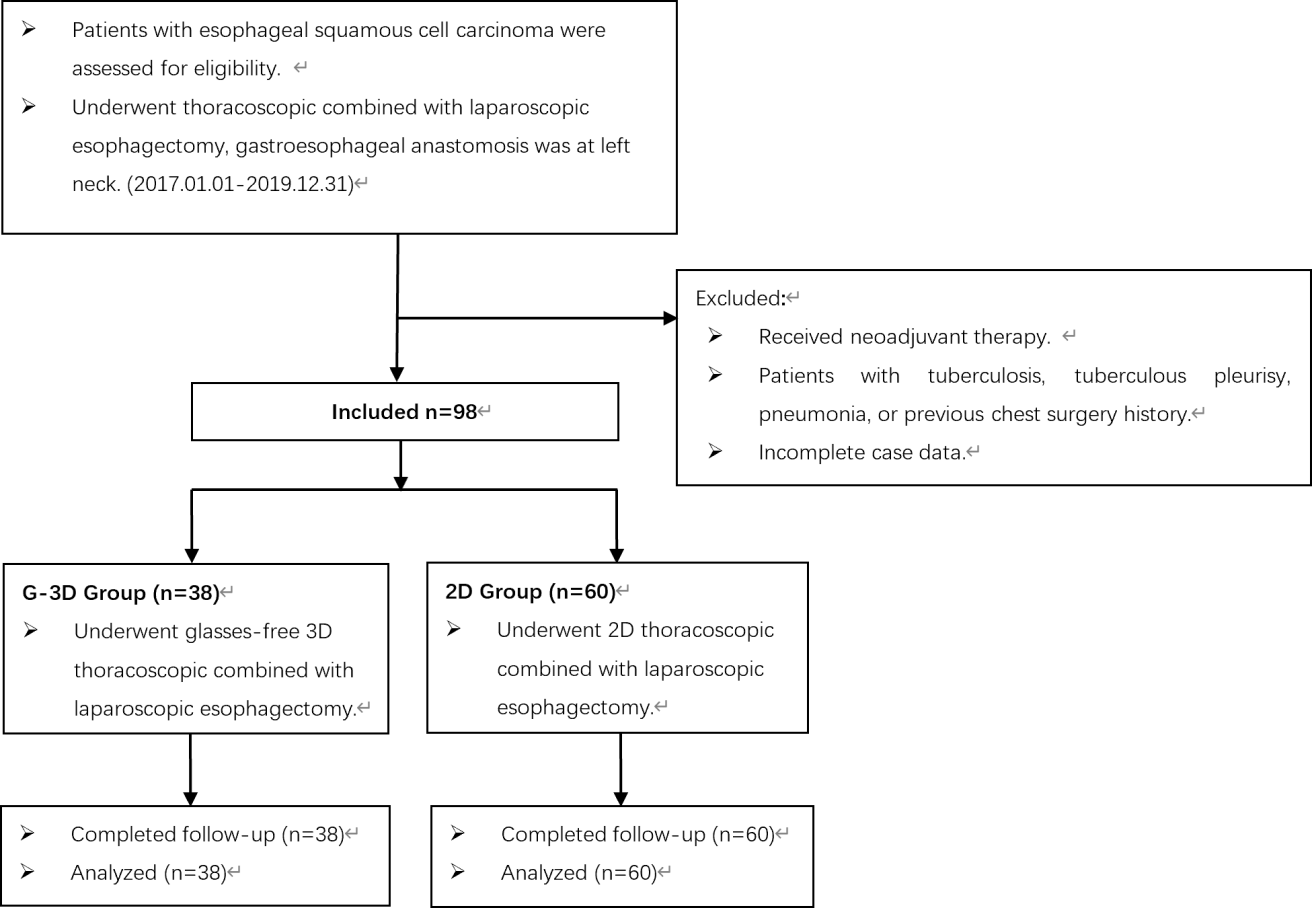
Flow chart summarizing the selection of eligible patients.

The inclusive criteria were as follows: (1) esophageal squamous cell carcinoma diagnosed by endoscopy and confirmed by pathology and (2) clinically evaluated as resectable esophageal cancer, and underwent thoracoscopic combined with laparoscopic esophagectomy; gastroesophageal anastomosis was at the left neck. The exclusion criteria were as follows: (1) patients had received neoadjuvant therapy; (2) patients with tuberculosis, tuberculous pleurisy, pneumonia, or previous chest surgery history; and (3) incomplete case data. Based on the above criteria, a total of 98 patients from January 2017 to December 2019, aged 45–77 years, were included in this study. According to the different thoracoscopic equipment, the patients were divided into two groups: the glasses-free 3D thoracoscopic group (G-3D group, 38 cases, including 30 men and 8 women), and the 2D thoracoscopic group (2D group, 60 cases, including 51 men and 9 women). The operation methods were as follows: the G-3D group was treated with a glasses-free 3D thoracoscopic system (Zhuhai Mingyi Medical Technology Co. Ltd. glasses-free 3D display, smv28f-g01) combined with laparoscopy for minimally invasive esophagectomy; gastroesophageal anastomosis was at left neck. The 2D group was treated with a 2D thoracoscopic system (German Storz 2D thoracoscopic system) combined with laparoscopy for minimally invasive esophagectomy; gastroesophageal anastomosis was at left neck. In the selection of glasses-free 3D or 2D thoracoscopic surgery, we adopt the principle of rotation, with a ratio of approximately 1:2, and try to ensure that the conditions of cases between groups are similar. A flow chart summarizing the selection of eligible patients is shown in **Figure 1**. The patient characteristics of the two groups are shown in **Table 1**.

**Table 1:** Summary of patient characteristics [ $\bar{x}$ ± *s*/(%)].

	G-3D Group (n=38)	2D Group (n=60)	P-value
Age	65.13 ± 6.55	62.55 ± 7.99	0.099
Sex			
Male	30 (78.9)	51 (85.0)	0.441
Female	8 (21.1)	9 (15.0)	
BMI (kg/m^2^)	23.62 ± 3.61	22.48 ± 3.13	0.101
ASA			
I	22 (57.9)	39 (65.0)	
II	13 (34.2)	17 (28.3)	0.492
III	3 (7.9)	4 (6.7)	
Smoking history	11 (28.9)	20 (33.3)	0.649
Drinking history	9 (23.7)	16 (26.7)	0.741
Concomitant diseases			
Hypertension	8 (21.1)	13 (21.7)	0.942
Diabetes	5 (13.2)	6 (10.0)	0.877
Coronary heart disease	3 (7.9)	6 (10.0)	1.000
Tumor location			
Upper	5 (13.2)	5 (8.3)	
Middle	19 (50.0)	32 (53.3)	0.687
Lower	14 (36.8)	23 (38.3)	
Pathological stage			
I	11 (28.9)	17 (28.3)	
II	14 (36.8)	21 (35.0)	0.800
III	12 (31.6)	19 (31.7)	
IV	1 (2.6)	3 (5.0)	
Adjuvant chemotherapy after operation	27 (71.1)	43 (71.7)	0.948

### Surgical procedure

The G-3D group was given intravenous-inhalation anesthesia and single lumen intubation. Thoracic part of MIE need to establish artificial pneumothorax of right thoracic cavity to get the surgical field. The surgeon did not need to wear 3D glasses, only wearing a surgical cap with a signal-receiving piece, while assistants need to wear 3D glasses. The patient’s position was left anteversion 30° prone position. One 1-cm incision was made in the 7th intercostal space of the axillary midline as the observation port. The incisions of approximately 1.0, 0.5, and 0.5 cm were made at the fourth intercostal space of the anterior axillary line, the sixth intercostal parascapular intercostal space, and the ninth intercostal space of posterior axillary line, respectively, which were operating ports. The lymph nodes were dissected under the carina, para-esophageal, trachea and bronchus, right recurrent laryngeal nerve (RLN), and left RLN. After thoracoscopic esophagectomy, a thoracic drainage tube [28F polyvinyl chloride (PVC) rigid thoracic drainage tube] was placed into thorax through the observation port. After the thoracic incisions were sutured, the patient was changed to a supine position; then, the stomach was dissociated by laparoscopy. Next, the cervical esophagus was dissociated and severed; the dissociative stomach was taken out through a small incision in the upper abdomen to make a tube stomach. The tube stomach was lifted to the neck through an esophageal bed path for end-to-side esophagogastrostomy (instrument anastomosis). Jejunostomy was performed by the small incision in the upper abdomen; the surgeon and assistants in the 2D group did not need to wear 3D glasses. The anesthesia method and operation procedure were the same as the G-3D group.

To investigate the factors associated with glasses-free 3D and 2D thoracoscopic MIE, we evaluated perioperative clinicopathological data, including age, sex, Body Mass Index (BMI),, American Society of Anesthesiologists (ASA) criteria,, smoking history, drinking history, concomitant disease, tumor location, pathological stage, thoracic operation time, thoracic intraoperative blood loss, surgeon’s uncomfortable feeling (including surgeon dizziness, surgeon asthenopia, and visual ghosting), total number of harvested lymph nodes, thoracic indwelling time, postoperative hospital stay, and postoperative complications(including severe lung infection, arrhythmia, anastomotic leakage, chylothorax, and recurrent laryngeal nerve injury).

### Statistical methods

Statistical analysis was performed using SPSS version 22.0 (SPSS Inc., Chicago, IL, USA). Data are presented as mean ± SD. Comparisons were made between the two groups using Student’s t-test for continuous measures and the chi-square test, Fisher’s exact test, or Mann–Whitney U test for categorical variables. Significance was set as a P-value < 0.05.

## Results

**Figure 2:**
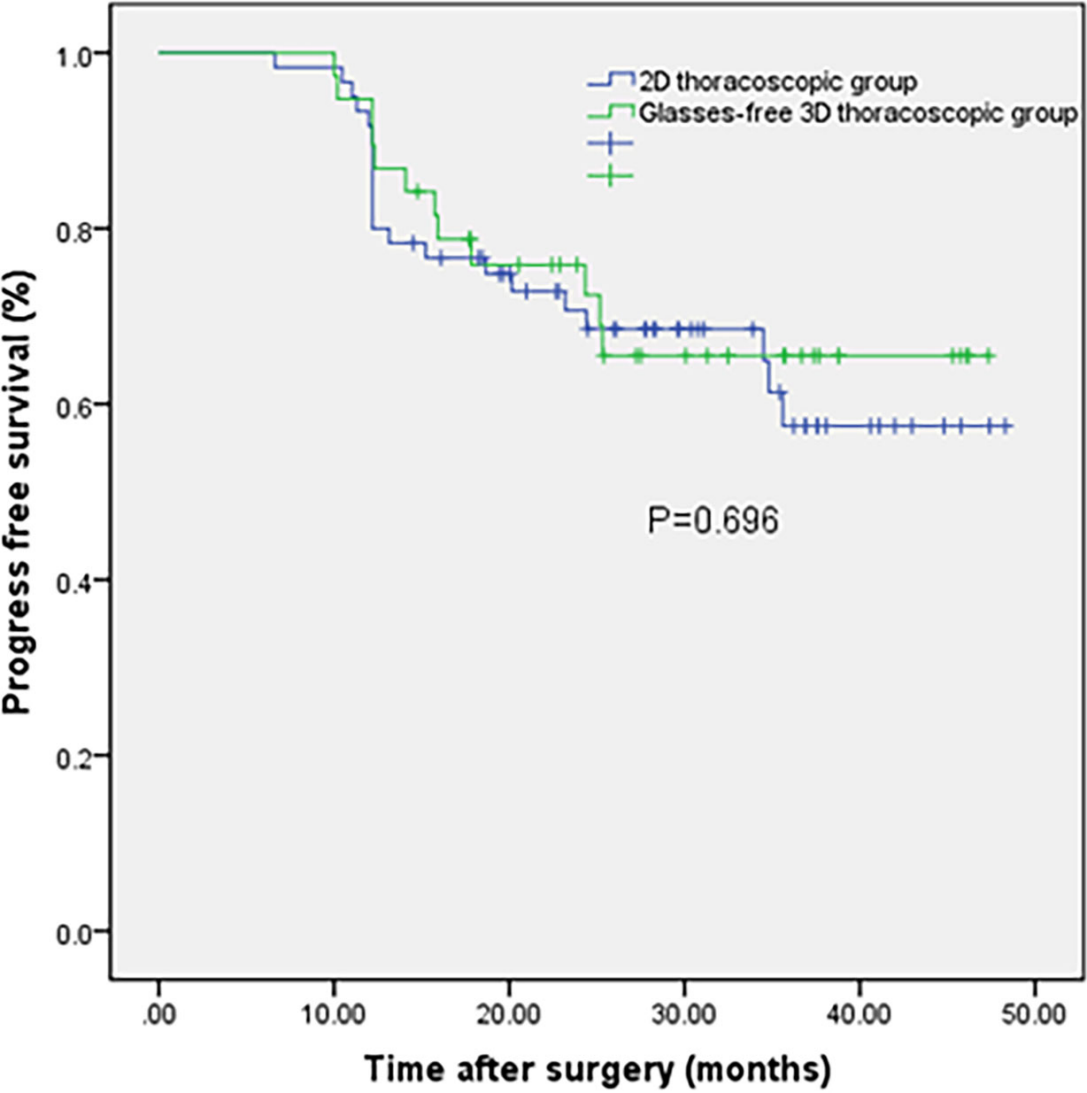
Kaplan–Meier curves for the glasses-free three-dimensional thoracoscopic group and two-dimensional thoracoscopic group.

Both groups of patients were successfully completed the operation.The surgical procedures in all 98 patients were relatively smooth, without conversion to thoracotomy,serious intraoperative bleeding, and intraoperative death cases. About the surgeon’s uncomfortable feeling, including surgeon dizziness, surgeon asthenopia, and visual ghosting, there were no significant differences between the two groups (P > 0.05). The thoracic operation time in the G-3D group was shorter than that in the 2D group [(75.45 ± 11.80) min vs. (88.15 ± 16.08) min, P = 0.000]. The total number of harvested lymph nodes in the G-3D group was more than that in the 2D group [(15.05 ± 2.66) vs. (12.40 ± 1.98), P = 0.000]. There was no significant difference in intraoperative blood loss and postoperative hospital stay between the two groups(P>0.05). There was no significant difference in the thoracic indwelling time, severe lung infection, arrhythmia, anastomotic leakage, chylothorax, and recurrent laryngeal nerve injury between the two groups (P > 0.05). There was no perioperative death in both groups. The outcomes of the two groups are shown in **Table 2**. The 1-year, 2-year, and 3-year progression-free survival (PFS) of the G-3D group were 89.5%, 57.9%, and 28.9%, respectively; and the 1-year, 2-year, and 3-year PFS of the 2D group were 80.0%, 55.0%, and 30.0%, respectively. There was no significant difference between the two groups on PFS (*P*>0.05). The survival time of the two groups is shown in **Table 3** and **Figure 2**.

**Table 2:** The outcomes of two groups [ $\bar{x}$ ± *s*/(%)].

	G-3D Group (n=38)	2D Group (n=60)	P-value
Surgeon dizziness	4 (10.5)	2 (3.3)	0.310
Surgeon asthenopia	4 (10.5)	3 (5.0)	0.527
Surgeon visual ghosting	2 (5.3)	0	0.148
Thoracic operation time (min)	75.45 ± 11.80	88.15 ± 16.08	0.000
Thoracic intraoperative blood loss (ml)	34.61 ± 6.62	36.92 ± 7.14	0.111
Total number of lymph nodes dissection	15.05 ± 2.66	12.40 ± 1.98	0.000
Thoracic indwelling time (d)	5.45 ± 0.98	5.62 ± 0.92	0.389
Postoperative complications			
Severe pulmonary infection	3 (7.9)	4 (6.7)	1.000
Arrhythmia	1 (2.6)	2 (3.3)	1.000
Anastomotic leakage	4 (10.5)	6 (10.0)	1.000
Chylothorax	2 (5.3)	3 (5.0)	1.000
Recurrent laryngeal nerve injuries	3 (7.9)	5 (8.3)	1.000
Perioperative death	0	0	–
Postoperative hospital stay (d)	15.24 ± 10.51	15.92 ± 10.37	0.754

**Table 3:** The survival time of two groups (%).

	G-3D Group (n=38)	2D Group (n=60)	P-value
Progression-free survival (PFS)			
1 year	34 (89.5)	48 (80.0)	0.216
2 year	22 (57.9)	33 (55.0)	0.778
3 year	11 (28.9)	18 (30.0)	0.911

## Discussion

### Whether the glasses-free three-dimensional thoracoscopy could settle the problems that two-dimensional or three dimensional cannot?

Esophageal cancer is one of the most common malignant tumors in China, and its main treatment is still surgical operation. Traditional thoracotomy and laparotomy for radical esophagectomy are limited in clinical application due to the adverse factors such as large trauma, high risks, and many postoperative complications (13–15). In recent years, the popularization of a minimally invasive concept, the wide application of endoscopic technology, and the establishment of an evidence-based medicine model have provided a safer and more feasible technical means for esophageal cancer surgery (16–19). The commonly used minimally invasive surgical methods include: 2D or 3D thoracoscopy combined with laparoscopic esophagectomy, Da Vinci robotic esophagectomy, and modified inflatable mediastinoscopy combined with laparoscopic esophagectomy (8, 20, 21). At present, 2D thoracoscopy has become the mainstream mode of thoracic surgery; however, due to its lack of depth and space sense, it is relatively easy to cause accidental injury and increase the difficulty of operation. Some domestic hospitals equipped with 3D thoracoscopy can solve this problem well. The surgeon wearing 3D glasses can effectively observe the 3D structure of the target tissues and organs. When performing some fine operations, such as cleaning the lymph nodes in the narrow tissue space, the relationship between the peripheral blood vessels and nerve tissues can be well observed, which can effectively avoid accidental injury and bleeding. However, because of the long operation time and special imaging technology of 3D, some surgeons wearing 3D glasses for a long time are prone to dizziness, asthenopia, visual ghosting, or other discomfortable symptoms during performing thoracoscopic esophagectomy, which affects the overall fluency of the surgical procedures. In addition, 3D glasses are black transparent polarized glasses, which significantly reduce the screen brightness and affect the visual field observation effect (22, 23). The question is: could glasses-free 3D thoracoscopy settle the above problems? In this study, we compared the clinical application of glasses-free 3D thoracoscopy and 2D thoracoscopy in esophageal cancer resection and found that it has some unique advantages.

### Glasses-free three-dimensional technology makes the operation more precise, which makes the operation safer

The results showed that there were significant differences in the operation time and number of lymph node dissection between the G-3D group and the 2D group (P < 0.05). In terms of lymph node dissection, at least 12 regional lymph nodes were dissected according to the Union for International Cancer Control (UICC) 7th edition of TNM (Tumor,Node and Metastasis) staging and diagnosis and treatment specifications. The total number of lymph nodes in the G-3D group and 2D group was (15.05 ± 2.66) vs. (12.40 ± 1.98), which met the standard, but the difference was statistically significant (P<0.01).We believe that glasses-free 3D thoracoscopy makes use of its unique depth effect, 3D imaging effect and approximately 20 times magnification visual effect technology to help the surgeon observe the blood vessels and lymph nodes more intuitively and clearly during the operation, which can avoid accidental injury. When dissecting the lymph nodes of the right recurrent laryngeal nerve, the surgeon can carefully identify the recurrent laryngeal nerve and the surrounding vena cava under glasses-free 3D thoracoscopy, which can clean the lymph nodes boldly, avoid injury, and reduce postoperative complications to a certain extent. For some patients with more peripheral fat or pleural adhesion, it has obvious advantages. When dissociating some blood vessels such as the azygous vein and left gastric artery, it can more accurately remove the perivascular fat tissue and omental tissue. For some patients with extensive pleural adhesion, it can separate the adhesions from multiple angles to avoid the injury of lung parenchyma and other main organs. Compared with 2D thoracoscopy, the operation of glasses-free 3D thoracoscopy is easier, and it is easier to grasp, separate, and cut under the thoracoscopy when using the instruments for the first time, which greatly shortens the learning curve and makes the operation of eye–hand cooperation more coordinated (24, 25). Most importantly, glasses-free 3D technology completely gets rid of our commonly used 3D glasses. Before surgery, the chief surgeon only needs to stick the pad with tracking marks on the surgical cap and can capture the surgical field on the glasses-free 3D thoracoscopic display without glasses at all. Moreover, the visual effect is bright and clear, without dullness and obvious ghosting, which minimizes the dizziness and ghosting caused by 3D glasses, making it easier for the chief surgeon to quickly adapt to the 3D effect, reducing discomfort and making the operation safer. About the surgeon’s uncomfortable feeling in this study, the chief surgeon had dizziness in four cases, had asthenopia in four cases, and had visual ghosting in two cases in the G-3D group, but the chief surgeon only had asthenopia in three cases in the 2D group. Although the number of uncomfortable symptoms of the surgeon of the G-3D group was at least twice that of the 2D group, there were no significant differences between the two groups. The chief surgeon said that the above uncomfortable symptoms did not affect the smoothness of the operation. It can be said that glasses-free 3D thoracoscopy is superior to 2D and 3D in some respects.

### Glasses-free three-dimensional thoracoscopic esophagectomy does not increase postoperative complications and has similar postoperative survival time to two dimensional

In our study, there was no significant difference in postoperative thoracic drainage time, severe pulmonary infection, arrhythmia, anastomotic leakage, right recurrent laryngeal nerve injury, and other complications between the two groups. The incidence of anastomotic leakage in the two groups (13.3% and 11.4%) was consistent with the reports from many domestic and foreign clinical centers (18, 19, 26). Therefore, glasses-free 3D thoracoscopic esophagectomy did not increase the incidence of postoperative complications. In clinical follow-up, most of the cases were still alive, so we took tumor recurrence or death as the endpoint, that is, we followed up the PFS time of patients. In our study, the 1-year, 2-year, and 3-year PFS of the G-3D group were 89.5%, 57.9%, and 28.9%, respectively; and the 1-year, 2-year, and 3-year PFS of the 2D group were 80.0%, 55.0%, and 30.0%, respectively. The difference between the two groups on PFS was not statistically significant, and long-term outcomes should be followed up.

### Some problems of glasses-free three-dimensional thoracoscopic surgery

The glasses-free 3D system requires that a tracking marker be attached to the surgeon’s cap, and the surgeon should stand facing the display screen. Two intelligent cameras above the display screen of the glasses-free 3D system recognize the marker and start working immediately. The cameras can intelligently determine the position of the left and right pupils of the surgeon according to the real-time captured images and then display the 3D images on the screen where the surgeon’s eyes are focused within the range of 1.4–2 m. According to reports, the response time of this glasses-free 3D system is less than 0.01 ms. It can track the eyes of the surgeon at any time, and make immediate adjustments according to the artificial intelligence (AI) algorithm, always ensuring the accuracy, fullness, and stereo of the images. With the help of this system, the surgeon’s vision is wider, the operation area is clearer, the surgeon’s eyes are not as tired as before, and the operation efficiency and accuracy are also improved. However, we also found some problems during the operation of glasses-free 3D thoracoscopic esophagectomy in our study: (1) the proper position of the display screen and surgeon should be adjusted in advance, and the distance should not be too close or too far; otherwise, the surgeon would see an obvious double shadow, which would affect the operation, so it was necessary for the surgeon to choose the best visual effect position and kept the display screen fixed before the operation. (2) During the operation,surgeon interacted with the glasses-free 3D system through a tracking marker on his cap, without wearing 3D glasses, and the surgical assistants needed to wear 3D glasses, so they could not share the glasses-free 3D visual effect. (3) The chief surgeon had dizziness, asthenopia, and visual ghosting in some cases of the G-3D group, but the uncomfortable symptoms did not affect the operation. The above problems may be related to the design and manufacturing issues of glasses-free 3D devices, the stability of the glasses-free 3D system, or the unskilled operation of the mirror assistant. We believe that the glasses-free 3D system still needs to be continuously innovated to meet the clinical needs.

### Glasses-free three-dimensional thoracoscopic esophagectomy may have a bright future

With the rapid development of thoracoscopic technology and the continuous renewal of the concept of accelerated rehabilitation surgery, the application of related research and technology in thoracic surgery is getting wider and wider. It is clear that 3D technology brings surgeons a new surgical experience and ultimately benefits patients. However, wearing 3D glasses for a long time will inevitably have some disadvantages, such as dark light, facial discomfort, and easy visual fatigue. Sometimes, the air exhaled by the surgeons forms fog on the glasses. Glasses-free 3D thoracoscopy, which depends on the glasses-free 3D system, makes MIE more precise, can help surgeons to complete the operation more safely and effectively, reduce surgeons’ psychological burden, relieve surgeons’ visual asthenopia, and ensure patients’ safety during the operation. Although the domestic technology still has some defects, we believe that glasses-free 3D thoracoscopy will be an important direction of surgery in the future, which is worthy of clinical application and further exploration.

## Conclusions

Our study shows that glasses-free 3D thoracoscopic MIE for esophageal cancer has higher safety, more lymph node dissection, and higher operation efficiency through the optimized surgical operations. Glasses-free 3D thoracoscopic surgery for esophageal cancer is a safe and effective surgical procedure. However, it must be pointed out that the study is a retrospective study, and its conclusion has certain limitations due to historical or selective factors. We believe that glasses-free 3D thoracoscopy for MIE is worthy of clinical promotion and further research should be initiated and implemented.

## Data availability statement

The original contributions presented in the study are included in the article/**Supplementary Material**. Further inquiries can be directed to the corresponding authors.

## Ethics statement

Written informed consent was obtained from the individual(s) for the publication of any potentially identifiable images or data included in this article.

## Author contributions

TH and HN conceived and designed the study. WR, DX, CZ, XN, LC, and FY collected the data. WR, DX, and XZ analyzed the data. WR wrote the first draft. All authors contributed to the article and approved the submitted version.

## Funding

This study was supported by the Project of youth program of shanghai health and family planning commission(20154y0040), the project of general program of national natural science foundation (81470213),youth program of national natural science foundation (81402449).

## Conflict of interest

The authors declare that the research was conducted in the absence of any commercial or financial relationships that could be construed as a potential conflict of interest.

## Publisher’s note

All claims expressed in this article are solely those of the authors and do not necessarily represent those of their affiliated organizations, or those of the publisher, the editors and the reviewers. Any product that may be evaluated in this article, or claim that may be made by its manufacturer, is not guaranteed or endorsed by the publisher.
